# Bioactive Chalcones from *Aizoon africanum*: Isolation and Cytotoxicity Against Liver and Neural Cancer Cells

**DOI:** 10.3390/plants14152389

**Published:** 2025-08-02

**Authors:** Ali O. E. Eltahir, Naeem Sheik Abdul, Taskeen F. Docrat, Paolo Bristow, Elias Chipofya, Robert C. Luckay, Monde A. Nyila, Jeanine L. Marnewick, Kadidiatou O. Ndjoubi, Ahmed A. Hussein

**Affiliations:** 1 Department of Chemistry, Cape Peninsula University of Technology, Bellville 7535, South Africa; aliomers250@gmail.com (A.O.E.E.); dickakadi@yahoo.fr (K.O.N.); 2 Department of Biochemistry, Stellenbosch University, Stellenbosch 7600, South Africa; sheikn@sun.ac.za (N.S.A.); 22304312@sun.ac.za (P.B.); 3 Applied Microbial and Health Biotechnology Institute, Cape Peninsula University of Technology, Bellville 7535, South Africa; docratt@cput.ac.za (T.F.D.); eliaschipofya@gmail.com (E.C.); marnewickj@cput.ac.za (J.L.M.); 4 Department of Chemistry and Polymer Science, Stellenbosch University, Matieland, Stellenbosch 7600, South Africa; rcluckay@sun.ac.za; 5 Department of Life and Consumer Sciences, College of Agriculture and Environmental Sciences, University of South Africa, Pretoria 1710, South Africa; nyilama@unisa.ac.za

**Keywords:** *Aizoon africanum*, phenolic compounds, flavanone, chalcone, dihydrochalcone

## Abstract

*Aizoon africanum* (L.) Klak (Synonym *Galenia africana* L.) is traditionally used for a variety of medicinal purposes; however, it has been reported to cause liver damage and severe ascites, particularly in sheep and Angora goats in the arid regions of the Western Cape. This study explores its cytotoxic properties to identify potential cytotoxic compound(s) in the plant. The methanolic extract of *A. africanum* was re-investigated and subjected to various chromatographic techniques, including preparative HPLC, resulting in the isolation of eight compounds (**1**–**8**). Structural elucidation was primarily based on NMR data. Among the isolated compounds, four were flavanones, one was a flavonone, and three were chalcones. Notably, compound **8** was identified as a new chalcone, while compounds **2** and **3** were reported for the first time from this plant. The toxicity of these isolated compounds was evaluated against the HepG2 and SH-SY5Y cancer cell lines using the MTT assay. We further investigated markers of cell death using spectrophotometric and luminometric methods. Among the isolated compounds, **7** and **8** exhibited cytotoxic activities within the range of 3.0–20.0 µg/mL. Notably, the compounds demonstrated greater cytotoxicity towards liver-derived HepG2 cells compared to the neuronal SH-SY5Y cell line. Compound **7** (2′,4′-dihydroxychalcone) was identified as inducing apoptosis through the intrinsic pathway without causing overt necrosis. The findings indicate that the phytochemicals derived from *A. africanum* exhibit differential cytotoxic effects based on cell type, suggesting potential for developing novel anticancer agents, particularly compound **7**. Additionally, the identification of compound **8** provides insight into the liver toxicity of this plant observed in sheep in South Africa.

## 1. Introduction

*Aizoon africanum* (L.) Klak (Synonym *Galenia africana* L.), belongs to family Aizoaceae and native to the southern region of African continent Angola, South Africa, Free States and Namibia [1]. In South Africa, it is known as “kraalbos,” with a history of use as a traditional medicine. It has been used to treat various ailments, including skin issues, coughs, and respiratory conditions, the oral route of administration is not documented in the literature; however, indigenous sources report that excessive use can cause blistering of the oral mucous membranes [2]. Scientific studies have supported its antimicrobial, anti-mycobacterial, and anti-fungal properties [3,4].

The plant’s safety is of major concern, categorised as a hazard to humans and livestock. The plant is linked to liver damage and the development of severe ascites, known as “waterpens” or “water belly,” especially in sheep and angora goats in the arid regions of the western cape [5]. The clinical and pathological characteristics indicate that *A. africanum* primarily affects the liver, with myocardial involvement observed only in the later stages of intoxication. Toxicological studies on the plant suggest that it may accumulate harmful levels of both oxalates and nitrates [6].

Previous phytochemical studies resulted in the isolation of different metabolites, including different classes of flavonoids, a chromone, and an amine [7,8,9] Additionally, more compounds were detected using LC-MS, mainly flavonoids [2].

There are a few studies on the cytotoxic activity of the extract and/or its compounds. One study by Mohamed et al., (2020) investigated the anticancer activity of an ethanolic extract against oestrogen receptor-positive (MCF-7) and triple-negative (MDA-MB-231) breast cancer cells [1]. The extract effectively inhibited cancer cell growth migration and induced oxidative stress and DNA damage, leading to cell cycle arrest and programmed cell death through multiple pathways, including apoptosis, necroptosis, and autophagy. These findings suggest that the whole extract of *A. africanum* has potential as an anticancer agent, and further in vivo research is warranted [2,10]. Another study involved human skin cells (HaCaT) and malignant melanoma cells (A375) [11]. *A. africanum* exhibited a significant dose- and time-dependent reduction in the viability of A375 cells, with no discernible impact on HaCaT cells. The A375 cells showed characteristics such as nuclear condensation, brightly stained nuclei, and nuclear fragmentation, indicative of apoptosis [11]. These findings suggest a clinical rationale for considering *A. africanum* as a potential anti-melanoma agent, providing efficacy with low toxicity. These studies suggest that the phytochemicals present in *A. africanum* act in synergism to augment overall mechanisms of action; however, analysis focusing on a single compound is likely to show better correlation between its concentration and induced toxicity profiles. This could provide valuable insights and exploration into novel lead compounds and design optimisation for anticancer drug development.

There is little information about the cytotoxic activity of *A. africanum* chemical constituents, primarily related to the liver and brain. While the crude extracts of *A. africanum* have been acknowledged for their toxic effects, there remains a dearth in knowledge concerning the specific toxicity and cell death mechanisms associated with its isolated compounds. This study explored the biological activity of compounds isolated from *A. africanum* by assessing their cytotoxicity against the human derived hepatocellular carcinoma (HepG2) and neuroblastoma (SH-SY5Y) cell lines. These cell lines were selected as relevant due to the well-established hepatotoxicity and potential neurotoxicity associated with *A. africanum* consumption, which suggests an innate bioactivity that could be leveraged therapeutically. These in vitro models will therefore provide crucial insight into the therapeutic value of isolated compounds against liver and brain-derived cancers, leading to the identification of promising anticancer leads with defined pharmacological properties.

## 2. Results

### 2.1. Chemical Study of a Methanolic Extract of A. africanum

**Figure 1:**
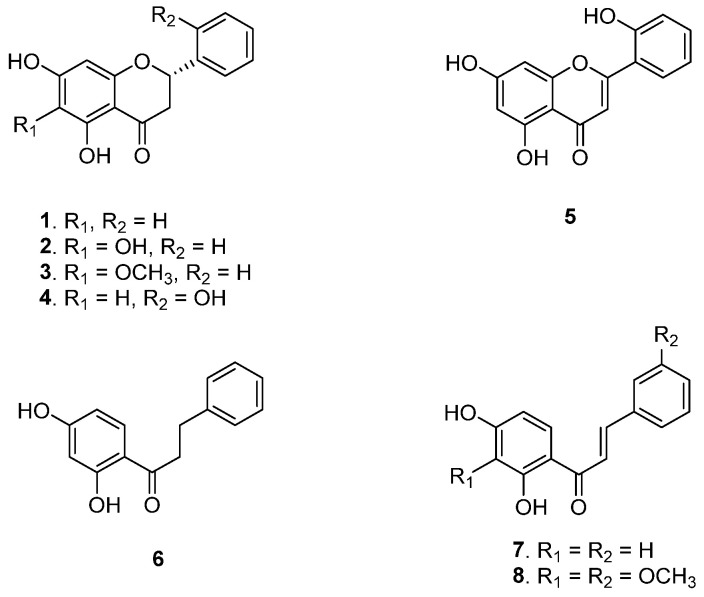
Chemical structures of compounds **1**–**8** from *A. africanum*.

From *A. africanum* total extract, eight pure compounds (**1**–**8**, Figure 1) were isolated and identified, namely: 2(*S*)-5,7-dihydroxyflavanone (**1**) [4]; 2(*S*)-5,6,7-trihydroxyflavanone (**2**); and 2(*S*)-5,7-dihydroxy-6-methoxyflavanone (**3**), previously isolated from *Scutellaria baicalensis* [12]. and *Helichrysum cymosum* [13]. Compounds **2** and **3** are reported here for the first time from this plant. Additional compounds include 2(*S*)-2′,5,7-trihydroxyflavanone (**4**) [4,8]; 2′,5,7-trihydroxyflavone (**5**) [14,15]; 2′,4′-dihydroxydihydrochalcone (**6**) [4]; and 2′,4′-dihydroxychalcone (**7**) [8] all previously reported from the same source (Table S1, and Figures S1–S7). Notably, (*E*)-2′,4′-dihydroxy-3,3′-dimethoxychalcone (**8**) is reported here for the first time. As shown in Table 1, the NMR data of compound **8** in DMSO-*d*_6_ reveals significant spectral characteristics.

### 2.2. Antioxidant Activities

In the results, we observed significant variations in the antioxidant parameters among the different samples, with distinct trends in the FRAP, ABTS, and DPPH assays.

**Figure 2:**
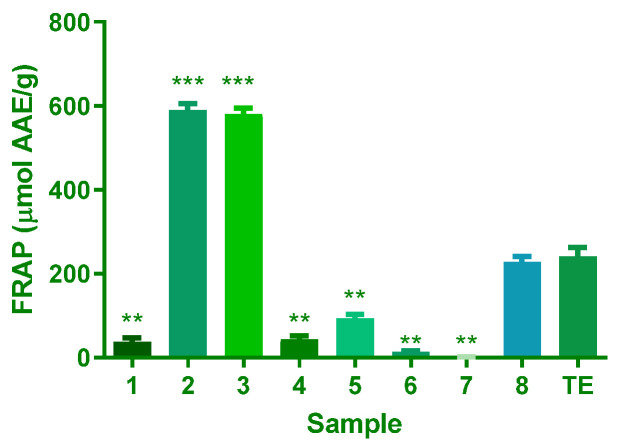
The ferric reducing antioxidant potential (FRAP, µM AAE/g) of samples isolated from *A. africanum*. Results are expressed as mean and SD of triplicate evaluations (n = 3). For the significant difference in compounds **1**–**8** vs. TE at *p* < 0.05, ** for *p* ≤ 0.01, and *** for *p* ≤ 0.001.

The FRAP assay measures compounds’ reducing ability, which indicates their potential to counteract oxidative stress. In a comparative analysis of antioxidant capacities, compound **1** had a significantly lower antioxidant capacity than the crude extract, with a mean difference of 203.2 µM AAE/g. Similarly, compounds **4**, **5**, **6**, and **7** also showed lower capacities, with mean differences of 197.3, 147.0, 227.4, and 239.7 µM AAE/g, respectively. In contrast, Compounds **2** and **3** exhibited higher antioxidant capacities, with mean differences of −349.4 and −339.1 µM AAE/g, respectively. Compound **8**’s antioxidant capacity was comparable to the crude extract. Data indicates that compound **2** consistently displayed the highest values at 590.3 µmol AAE/g. Conversely, compound **7** showed the lowest FRAP values, with 1.193 µmol AAE/g (Figure 2).

**Figure 3:**
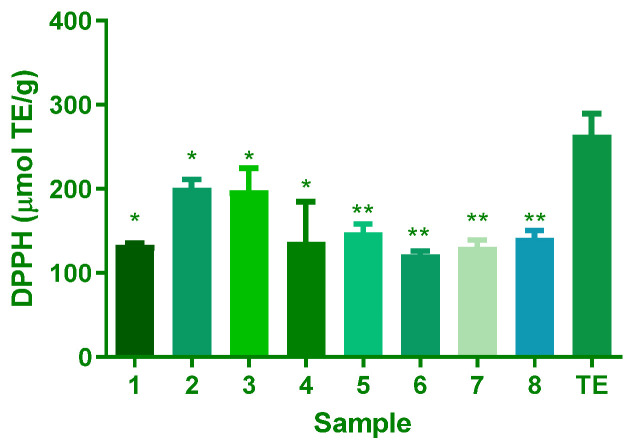
Antioxidant capacity of *A. africanum* isolates using the DPPH method (µmol TrE/g). Results are expressed as mean and SD of triplicate evaluations (n = 3). For the significant difference in compounds **1**–**8** vs. TE at *p* < 0.05, * for *p* ≤ 0.05 and ** for *p* ≤ 0.01.

The 2,2-Diphenyl-1-picrylhydrazyl (DPPH) assay assesses the ability of compounds to neutralise DPPH radicals. The DPPH assay results revealed that all eight isolated compounds (**1**–**8**) exhibited significantly lower antioxidant capacity than the crude extract. Specifically, compounds **1**, **4**, **5**, **6**, **7**, and **8** showed mean differences ranging from 115.7 to 142.3 µmol TrE/g, while Compounds **2** and **3** had minor but still significant differences of 62.65 and 66.11 µmol TrE/g, respectively (Figure 3). These results indicate that the synergistic interactions among multiple compounds within the crude extract likely contribute to its overall antioxidant potency.

**Figure 4:**
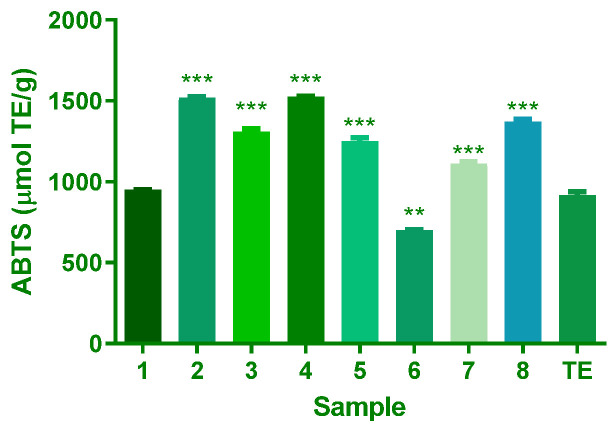
The relative antioxidant ability of isolates from *A. africanum* to scavenge the radical ABTS^+^. Results are expressed as Trolox equivalents (µmol TrE/g), mean, and SD of triplicate evaluations (n = 3). For the significant difference in samples 1–8 vs. TE at *p* < 0.05, ** for *p* ≤ 0.01, and *** for *p* ≤ 0.001.

The Trolox Equivalent Antioxidant Capacity (TEAC) assay is a valuable method for evaluating the antioxidant capacity of various compounds by measuring their ability to scavenge ABTS radicals. Compound **1** showed similar antioxidant capacity to the extract. Compounds **2**–**5**, **7**, and **8** demonstrated significantly higher antioxidant capacities, with mean differences of −603.2, −391.0, −607.6, −334.2, −194.4, and −452.9 µmol TrE/g, respectively. In contrast, compound **6** exhibited a lower antioxidant capacity with a mean difference of 217.2 µmol TrE/g (Figure 4).

### 2.3. Comparison of Inhibitory Activities of A. africanum Phytoconstituents on Cell Proliferation of HepG2 and SH-SY5Y

The HepG2 and SH-SY5Y cell lines are routinely used to determine potential anticancer agents’ cytotoxic effects. All the phytoconstituents isolated from *A. africanum* were more cytotoxic to HepG2 cells than the TE, except **1**, which maintained viability above 95% for all tested concentrations. The calculated results showed **7** as the most hepatic cytotoxic compound with an IC_50_ of 3.0 µg/mL. All results are shown in Table 2. The cytotoxicity of compounds targeted against neuroblastoma cells showed **8** as the most toxic one (Table 2).

Overall, the phytoconstituents isolated from *A. africanum* showed higher cytotoxicity against the liver carcinoma cell line when compared to the neuroblastoma cell line, and the isolated compounds were more potent in both cell lines than the TE.

Since the IC_50_ values of **7** and **8** were the most cytotoxic in HepG2 cells, further experiments were performed in this cell line to elucidate the mode of cell death.

### 2.4. Compound ***7*** Induced Apoptosis by Activation of Caspase-Dependent Pathways

The apoptosis signal transduction was investigated to elucidate the cell death mechanisms of **7** and **8** in HepG2 cells. Caspases play a crucial role in the initiation and execution of apoptosis. Caspases-8 and -9 are known as initiator caspases, whereas caspase-3 is considered as an executioner caspase.

**Figure 5:**
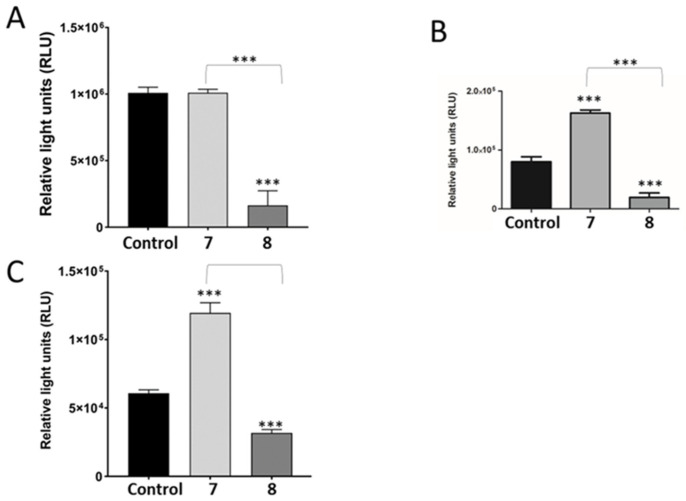
Test compounds significantly influenced apoptotic cell death markers. The activity of initiator caspase 8 (**A**) was significantly downregulated by compound **8**. Caspase 9 activity (**B**) was significantly elevated by **7** with a concomitant increase in the executioner caspase 3 activity (**C**), (n = 3). *** *p* < 0.001.

Caspase activity assays (Figure 5C) showed that **7** significantly increased the activity of caspase 3, while **8** significantly decreased caspase 3 activation compared to control cells. The two distinct and well-known initiator caspases, caspase-8 for the death receptor-mediated and caspase-9 for the mitochondria-mediated pathways, have been shown to initiate apoptosis. Therefore, the activity levels of caspases-8 and -9 were assessed.

Treatment with **7** did not alter caspase-8 activity, while **8** significantly decreased activity (Figure 5A). The activity of mitochondria-mediated apoptotic cell death initiator caspase-9 was significantly increased in **7** but not **8**. These data indicate that only compound **7** triggered the intrinsic pathway and activation of caspase 3 (Figure 5B).

### 2.5. Compound ***8*** Induced Necrosis as a Cell Death Mechanism

**Figure 6:**
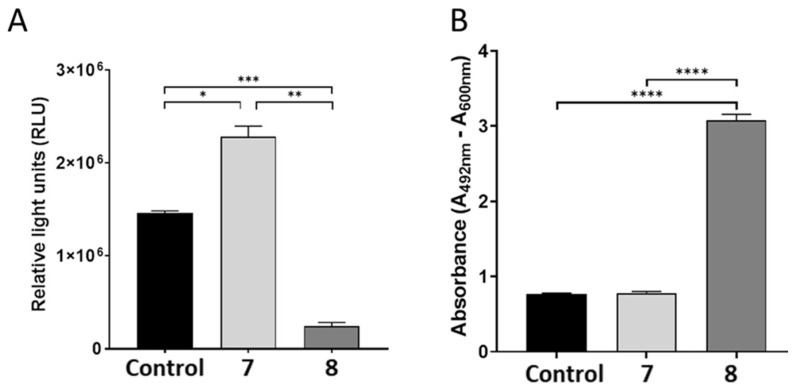
Intracellular ATP levels (**A**) of HepG2 cells significantly influenced by **7** and **8**. Compound **8** induced significant LDH leakage from HepG2 cells indicative of increased necrosis (**B**), (n = 3). **** *p* < 0.0001, *** *p* < 0.001, ** *p* < 0.01, * *p* < 0.05.

The apoptotic death signalling pathway contains several ATP-dependent steps, some of which might determine whether cell death occurs by apoptosis or necrosis, the possibility that treatment with **8** inhibited caspase activation because of ATP depletion was determined. The results show that **8** drastically decreased intracellular ATP levels while **7** improved ATP levels (Figure 6A).

A key signature for necrotic cells is the permeabilisation of the plasma membrane. This event can be quantified in cell culture settings by measuring the release of the intracellular enzyme lactate dehydrogenase (LDH). We showed that **8** induced significant necrotic cell death as indicated by the release of LDH when compared to **7** and control cells (Figure 6B).

### 2.6. Toxicological Profile

Toxicity predictions from the ProTox (ProTox 3.0; AG Preissner; Charite Berlin) platform indicated that all compounds (Table 3) were inactive for hepatotoxicity (probability: 0.56–0.68) and neurotoxicity (0.71–0.86). However, all were flagged for BBB (blood-brain barrier) toxicity (probability: 0.61) and showed a high likelihood of mitochondrial membrane potential (MMP) disruption (0.98–1.00), suggesting potential for mitochondrial-mediated toxicity relevant to both liver and CNS injury [16,17].

### 2.7. Physiological, Pharmacokinetic, Drug-Likeness and Lead-Likeness Properties

**Figure 7:**
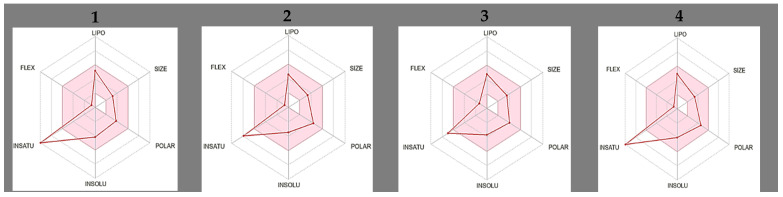

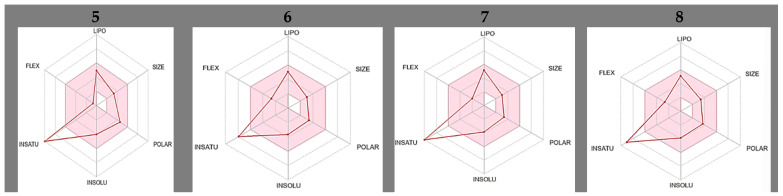
Physicochemical properties of compounds **1**–**8**.

All compounds displayed favourable drug-like profiles with molecular weights < 500 g/mol, acceptable lipophilicity (iLOGP: 1.89–3.08), and good water solubility (Log S: −3.49 to −4.03), with no P-glycoprotein (P-gp) substrate except compound **2**. The predicted GI absorption was high across all compounds, with compounds **1**, **3**, **6**, **7**, and **8** being BBB-permeable. CYP inhibition (CYP1A2, 2C9, 2C19, 2D6, 3A4) was observed in all compounds, indicating a risk of metabolic interactions. All compounds followed Lipinski’s Rule of Five, while only **6** and **7** failed to abide by all lead-likeness properties. The bioavailability score was 0.55 for all compounds, indicating moderate oral bioavailability (Figure 7).

## 3. Discussion

Fractionation and purification of phytochemicals from total methanolic extract using different chromatographic techniques, including HPLC, resulted in the isolation of eight pure compounds (**1**–**8**). Compound **8** was obtained as an amorphous powder, and its molecular formula, C_17_H_16_O_5_, was established by the HREIMS data observed at *m*/*z* 301.1068 (corresponding to C_17_H_17_O_5_, [M+H]^+^). The FTIR spectrum showed absorption peaks at 3400, 2934, 1649, 1583, 1463, 1343, 1215, and at 1049 cm^−1^ can be assigned to OH, C=O groups and C-H, C=C, C-C, and C-O bonds.

**Figure 8:**
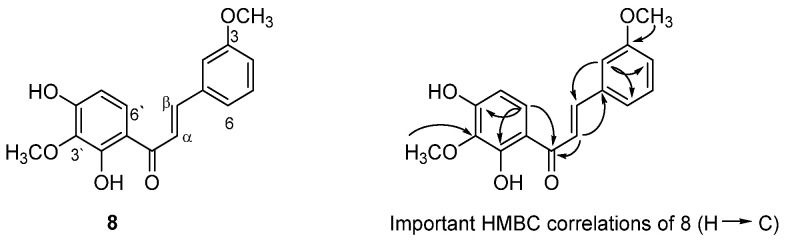
Chemical structure and selected HMBC correlations of **8**.

The detailed characterisation of compound **8** is further supported by the chemical structure and selected HMBC correlations presented in Figure 8, highlighting key molecule interactions. The ^1^H NMR spectrum of **8** indicated the presence of a 1,3-disubstituted benzene ring [δ_H_ 7.50 (*br s*, H-2), 7.00 (*br d*, 8.0 Hz, H-4), 7.37 (*br t*, 8.0 Hz, H-5), and 7.44 (*br d*, 8.0 Hz, H-6)], a 1,2,3,4-tetrasubstituted benzene ring [δ_H_ 6.51 and 8.01 (*d* each, 8.0 Hz, H-5′ and H-6′)], two *E*-olefinic protons (δ_H_ 7.97 and 7.87 (*d* each, 15.6 Hz, H-α and H-β), and two methoxy groups at 3.83/3.74 (Figure 8). The ^13^C NMR and DEPT-135 spectra showed 17 carbons, including four oxygenated carbons at δ_C_ 160.1 (C-3), 159.0 (C-2′), 135.2 (C-3′) and 158.4 (C-4′); two methoxy groups at δ_C_ 60.2 and 55.8; and 1,4-enone system at δ_C_ 192.4 (CO), 121.9 (C-α) and 144.2 (C-β).

The HMBC correlations showed cross peaks between (among others) H-2 and C-1, C-3, C-β, C-6, C-4; H-α/C-1, CO, C1′; and H-6′/C-4′, CO, C-2′ which confirm the connections among ring A and ring B carbons and with the 1,4-enone system of the chalcones structure. The positions of the methoxy groups at C-3 and C-3′ were confirmed by the HMBC correlation of the methoxy protons with the same carbons (Figure 8). Further, the NMR data are similar to (*E*)-3,2′,4′-trihydroxy-3′-methoxychalcone isolated from the same source [8]; the only difference is the presence of a methoxy group at C-3′ in **8**. The structure of **8** was deduced to be the new (*E*)-2′,4′-dihydroxy-3,3′-dimethoxychalcone.

The results of the antioxidant capacity test, comprising the polyphenols, FRAP, ABTS, and DPPH assays, offer valuable insights into the antioxidant potential of the *A. africanum* isolated compounds under examination. These assays are essential for evaluating substances’ ability to counteract free radicals and oxidative stress, pivotal factors in various diseases and ageing processes. The results indicate a significant difference in antioxidant capacity among the samples (Figure 2, Figure 3 and Figure 4). This highlights the importance of carefully evaluating plant extracts or compounds to determine their antioxidant potential. These variations are due to differences in the chemical structure of each compound. Significantly, **4** consistently demonstrated the highest values across multiple assays, indicating a robust antioxidant capacity. Moreover, **3** showcased notable results in the FRAP, ABTS, and DPPH assays, emphasising its potent antioxidant potential. These findings are of particular interest for the biological activity of the compound. In contrast, **6** consistently registered the lowest antioxidant values in all four assays.

The relationship between cytotoxicity and antioxidant potential is complex and context dependent. The assessment of the anticancer activity of plant extracts is crucial for safe treatment as the antioxidant profile of compounds from *A. africanum* may not be strictly cytoprotective but may instead reflect a redox-modulating capacity that contributes to its toxic effects. An in vitro cytotoxicity test using HepG2 and SHSY5Y cancer cell lines was performed to screen potentially toxic compounds that affect metabolic activity. The total extract (TE) showed growth inhibition effects on the two cancer cell lines, with **8** showing the greatest potency against HepG2 cells. The results also confirmed the differential impact induced by the phytochemicals against both cell lines. The inhibition of cell growth by *A. africanum* compounds might be due to the initiation of different cell death pathways. Previous studies suggested that TE induced mitochondrial and death receptor apoptosis and disrupted DNA integrity [2,11,18]. These studies were conducted using whole *A. africanum* extract. The mechanisms of cell death induced by isolated phytochemicals of *A. africanum* and its effects on liver cancer cells are not fully understood. Of the isolated compounds in this study, **7** (IC_50_ 3 µg/mL) and **8** (IC_50_ 24.14 µg/mL) were selected for further analysis of cell death markers.

The antioxidant and cytotoxic properties observed in this study, particularly for compounds **7** and **8**, may reflect an intricate and context-dependent interplay. Chalcones are known to act as redox modulators, capable of both scavenging reactive oxygen species (ROS) and, under certain conditions, generating ROS or disrupting redox-sensitive signalling pathways. This duality may explain why compounds exhibits both antioxidant activity and the ability to induce cytotoxicity. At lower concentrations, the hydroxyl groups likely contribute to ROS scavenging and redox balance, whereas at higher concentrations or in metabolically compromised cells, redox imbalance may lead to oxidative stress, activation of caspase pathways, and programmed cell death.

Apoptosis is a highly conserved process that can trigger various physiological and pathological conditions. Caspase activation leads to the proteolysis of several substrates, which finally results in the apoptotic collapse of the cell. To study whether compounds **7** and **8** induced apoptosis through caspase-dependent pathways, we analysed the activity of prominent initiator (8 and 9) and executioner caspases (3/7). Apoptosis occurs through intrinsic or extrinsic pathways. The extrinsic apoptosis pathway occurs after stimulation of death receptors that lead to activation of caspase 8 and subsequent activation of caspase 3/7. Caspase-9 is complexed with cytochrome C and Apaf-1, leading to the construction of the apoptosome complex and activation of caspase 3 [10]. As shown in Figure 4, only **7** initiated apoptosis through activating caspases 9 and 3, while compound **8** showed a significant decrease in all caspases tested.

Studies have suggested that ATP binding to Apaf-1 and ensuing hydrolysis are required for caspase-9 activation [10]. We show that **7** increased levels of ATP while **8** depleted intracellular ATP, thus providing energy for apoptotic cell death. Further analysis of cell death biomarkers revealed leakage of LDH due to loss of cell membrane integrity in cells treated with **8**. We speculate that the loss of membrane integrity aligns with our finding of decreased caspase 8 activity since death receptors needed to stimulate the extrinsic apoptotic pathway are damaged.

The physicochemical properties of the active compounds offer insights into their drug-likeness. Compound 7 is a dihydroxychalcone featuring two hydroxyl groups that may enhance aqueous solubility and facilitate interactions with biological targets, potentially supporting favourable oral bioavailability. In contrast, compound **8**, a dimethoxychalcone, is more lipophilic due to its methoxy substitutions, which may contribute to the observed increase in cytotoxicity.

Further in silico ADME (absorption, distribution, metabolism, and excretion) profiling indicated that compounds **7** and **8** inhibit key hepatic CYP enzymes (CYP1A2, 2C9, and 3A4), are not P-gp substrates, and are predicted to be both BBB permeable and BBB-toxic [19]. These properties suggest a heightened risk of CNS exposure and metabolic interactions. Both compounds also showed a high probability of mitochondrial membrane potential (MMP) disruption, indicating potential for neuro- and hepatotoxic effects.

Among them, compound **7** exhibited favourable lipophilicity (iLOGP = 2.23) and a moderate topological polar surface area (TPSA), supporting good membrane permeability (TPSA < 70 Å^2^ and iLOGP < 3). However, it failed lead-likeness criteria due to a molecular weight below 250 g/mol and an XLogP3 exceeding 3.5 [20]. Moreover, despite its low in silico toxicity predictions, compound 7 demonstrated strong in vitro cytotoxicity (HepG2 IC_50_ = 3.0 µg/mL; SH-SY5Y IC_50_ = 47.5 µg/mL), suggesting a false-negative result and highlighting the importance of validating computational predictions experimentally.

Neurotoxicity risk was also elevated in BBB-permeable, non–P-gp substrate compounds, with compounds **7** and **8** posing the highest overall risk of neuro- and hepatotoxicity, primarily due to CYP inhibition, BBB permeability, and mitochondrial disruption.

The scaffold of **7** is of interest due to the chalcone backbone’s established pharmacophore status in anticancer drug discovery [21]. The presence of hydroxyl groups at specific positions may be critical to its bioactivity, likely facilitating hydrogen bonding interactions with cellular targets. These hydroxyl groups also provide chemically accessible sites for further modification.

Future structure–activity relationship (SAR) studies could explore the impact of substituent variation at the hydroxyl positions, including methylation, halogenation, or replacement with electron-withdrawing or -donating groups, to tune lipophilicity, membrane permeability, and target selectivity. Such chemical optimisation could improve pharmacokinetic properties while retaining or enhancing apoptotic activity. Given the promising in vitro profile of **7**, it serves as a valuable lead compound for further medicinal chemistry development, including analogue synthesis, in silico docking, and mechanistic testing.

## 4. Materials and Methods

### 4.1. General

*A. africanum* was collected in May 2015 from the Western Cape Province, South Africa. Prof. Chris N. Cupido identified the plant; a specimen was deposited in Kirstenbosch National Botanical Garden (Cape Town, South Africa) under accession number 1468255/NBG. The plant was air-dried at room temperature. Silica gel 60 (0.063–0.200 mm particle size), Sephadex (LH-20), and aluminium thin layer chromatography (TLC) plates were supplied by Merck (Cape Town, South Africa). Analytical reagent (AR) grade solvent hexane, ethyl acetate, dichloromethane and methanol were purchased from Kimix (Cape Town, South Africa). HPLC grade methanol was used for purification procedures. HPLC Analysis chromatographic separations were conducted using a Shimadzu LC-20 high-performance liquid chromatography (HPLC) system equipped with a quaternary pump (LC-20AD), a diode array detector (DAD, SPD-M20A), and a manual injector. A reversed-phase C18 column (25 × 1 cm, 5 μm; Supelco, Merck, South Africa) was used. The DAD was set to monitor at 254, and 366 nm.

One-dimensional (^1^H, ^13^C, and DEPT-135) and two-dimensional (HMBC, HSQC, and COSY) NMR spectra were recorded on a Bruker spectrometer (Rheinstetten, Germany) operating at 400 MHz for ^1^H and 100 MHz for ^13^C. Deuterated solvents such as CDCl_3_, CD_3_OD, DMSO-*d*_6_, and acetone-*d*_6_ (Merck, South Africa) were used to dissolve the isolated compounds (Table S1 and Figures S1–S8). High-resolution ultra-performance liquid chromatography–mass spectrometry (UPLC–MS) analysis was performed using a Waters Synapt G2 quadrupole time-of-flight (QTOF) mass spectrometer coupled to a Waters Acquity UPLC system (Waters, Milford, MA, USA). The method and operating conditions were applied as described by Stander et al. [22]. Characteristic functional groups were recorded on a PerkinElmer Fourier-Transform Infrared Spectrometer 2000 equipped with a universal ATR-FTIR (PerkinElmer Spectrum 100, Llantrisant, Wales, UK) in the range of 400–4000 cm^−1^. Unless otherwise stated, chemical reagents for the biological assays were purchased from Merck, South Africa. Standards for the antioxidant assays were obtained from (Sigma-Aldrich, South Africa). Luminometric kits were sourced from Promega (Madison, WI, USA), whilst cytotoxicity kits were bought from Roche (Mannheim, Germany).

### 4.2. Extraction and Isolation

The dried plant material was extracted with methanol, a widely used solvent capable of extracting a broad range of polar compounds. The extraction was performed under heating at 60 °C to enhance efficiency. The resulting extract was then subjected to various chromatographic techniques, primarily size-exclusion chromatography using Sephadex, followed by semi-preparative HPLC. Due to the complexity of the plant’s constituents, purification could not be achieved using conventional open-column chromatography and required HPLC for effective isolation.

*A. africanum* (1.0 kg) was extracted using methanol with heating at 60 °C (4.0 L × 2 h × 2 times). The extract was filtered and concentrated under vacuum to yield the final extract of about 145.0 g. Part of the extract (50.0 g) was applied to a silica gel column chromatography, eluting with a hexane/ethyl acetate gradient mixture of increasing polarity up to 100% ethyl acetate. Similar fractions were combined according to the TLC profile to yield twenty-five major fractions.

**Figure 9:**
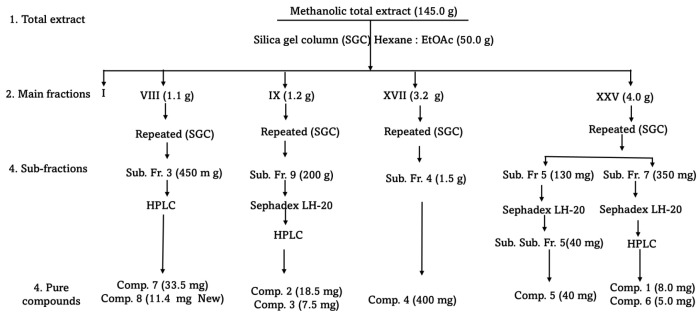
Chromatographic purification of *A. africanum* total extracts.

The main fraction VIII (1.10 g) was re-chromatographed on Sephadex developed with isocratic 20% aqueous ethanol followed by semi-prep HPLC using a gradient of MeOH: de-ionised water (DIW); (65:35, isocratic, flow rate, 1.0 mL/min) to give compounds **8** (11.4 mg, R_t_ 55–56 min) and **7** (33.5 mg, R_t_ 45–46 min). The main fraction IX (1.20 g) was re-chromatographed on Sephadex developed with isocratic 20% aqueous ethanol followed by semi-prep HPLC using a gradient of MeOH: de-ionised water (DIW); (65:35, isocratic, flow rate, 1.0 mL/min) to give compounds; **2** (18.4 mg, R_t_ 28–30 min) and **3** (7.5 mg, R_t_ 43–35 min). The main fraction XVII (3.20 g) was re-chromatographed on Silica, using gradient of hexane and ethyl acetate (0.0% to 50.0% EtOAc), to give compound **4** (400 mg). The main fraction XXV (4.0 g) was re-chromatographed on Sephadex developed with isocratic 20% aqueous ethanol followed by semi-prep HPLC using a gradient of MeOH: de-ionised water (DIW); (65:35, isocratic, flow rate, 1.0 mL/min) to give compounds; **1** (8.0 mg, R_t_ 26–27 min) and **6** (5.0 mg, R_t_ 43–44 min) from sub-fraction 7, compound **5** (40.0 mg, R_t_ 43–44 min) from sub-fraction 5: see Figure 9.

***E*-2′,4′-dihydroxy-3,3′-dimethoxychalcone** (C_17_H_16_O_5_): amorphous powder, IR: 3400 cm^−1^ (OH), 2934 cm^−1^ (CH), 1649 cm^−1^ (C=O), 1583, 1463, 1343, 1215, and at 1049 cm^−1^ (C=C, C-O) (Figure S10); UV: 260, 355 nm; HRESIMS: *m*/*z* 301.1068 [M+H]^+^ (calculated for C_17_H_17_O_5_, 301.1071); ^1^H and ^13^C NMR (DMSO-*d*_6_): see Table 1 (Figures S8 and S9).

### 4.3. Antioxidant Capacity Testing

#### 4.3.1. Ferric Reducing Antioxidant Capacity (FRAP)

The FRAP assay was conducted following the methodology outlined by Benzie and Strain [23]. In a 96-well transparent microplate, the sample was combined with the FRAP reagent in a 1: 30 µL ratio. The FRAP reagent (pH 3.6, Saarchem, Johannesburg, South Africa) consisted of 0.3 M acetate buffer 10 mM 2, 4, 6-tripyridyl-s-triazine (TPTZ) in 0.1 M HCl, and 20 mM iron (III) chloride hexahydrate (FeCl_3_·6H_2_O), all of which were diluted in 6.6 mL of distilled water. This mixture was incubated for 30 min at 37 °C in the microplate reader, allowing the FRAP reaction to occur. The absorbance was subsequently measured at 593 nm. We employed L-ascorbic acid as a standard, with concentrations spanning from 0 to 1000 μM. The results were quantified and expressed as μM ascorbic acid equivalents (AAE).

#### 4.3.2. Azinobis (3-Ethylbenzothiazoline-6-Sulfonate) (ABTS) Assay

The ABTS assay, based on [24], involved the preparation of stock solutions: 7 mM ABTS and 140 mM potassium-peroxodisulfate (K_2_S_2_O_8_). The working solution was created by mixing 88 μL of the K_2_S_2_O_8_ solution with 5 mL of the ABTS solution and allowing them to react (24 h, RT, in the dark). Trolox served as the standard, covering concentrations from 0 to 500 μM. The ABTS mix solution was diluted with ethanol to establish a start-up absorbance (control) of about 2.0 (±0.1). In the assay, 25 μL of the sample was incubated with 300 μL of ABTS (in the dark, RT, 30 min). Subsequently, a plate reader measured the absorbance at 734 nm at 25 °C. The results were expressed as μM Trolox equivalents (TE).

#### 4.3.3. DPPH

Following the methodology described by [25], we assessed the antioxidant potential of samples in scavenging DPPH radicals. Each sample and Trolox standard were subjected to dilutions, yielding concentrations of 0.08, 0.04, 0.02, 0.01, and 0.005 mg/mL, respectively. These diluted solutions were mixed in equal proportions (1:1) and thoroughly vortexed. Subsequently, the mixture was incubated at room temperature for 30 min. A 300 µL volume of this solution was then dispensed into a 96-well microplate, and its absorbance was measured at 517 nm. The study’s findings were expressed as micromoles of Trolox equivalent (µmol/TE), providing insight into the antioxidant capacity of the plant extract in neutralising DPPH radicals.

#### 4.3.4. Cell Culture

Selecting suitable cell lines was critical in assessing the cytotoxic effects of *A. africanum* phytochemicals. We opted to work with two distinct human cell lines, HepG2 and SHSY-5Y, each chosen for its specific relevance. HepG2 cells, derived from hepatocellular carcinoma, were selected as they represent a valuable model for liver cells [26]. These cells are widely employed in cytotoxicity assays due to their direct relevance to hepatotoxicity studies and drug screening. On the other hand, SHSY-5Y cells, derived from human neuroblastoma, were chosen as a neural cell model, providing insights into the potential neurotoxicity of the compounds under investigation. The human neuroblastoma (SH-SY5Y) and the human hepatocellular carcinoma (HepG2) cell lines were cultured in DMEM (ThermoFisher, 42430025, Johannesburg, South Africa,) containing 10% foetal bovine serum (FBS) and 5% anti-biotic/anti-mycotic (15240062, Thermo Fisher). Cells were maintained at 37 °C in a humidified incubator containing a 5% CO_2_ atmosphere.

##### Cell Viability Assay

To determine the effect of *A. africanum* phytoconstituents on cell viability, the methyl thiazol tetrazolium (MTT, Sigma-Aldrich, St Louis, MO, USA) assay was used. Approximately 15,000 cells were seeded into a 96-well microtitre plate (n = 3). The cells were treated with a concentration range of 0–500 μg/mL (in triplicate for 24 h) of the test compounds. This range was selected to ensure a complete dose–response and is consistent with literature showing this range effectively elicits measurable outcomes in various cell lines exposed to *A. africanum* [2,11]. Treatments were removed, and the cells were incubated with an MTT salt solution [5 mg/mL in 0.1 M phosphate-buffered saline (PBS, Lonza Group Ltd., Verviers, Belgium)] and media (4 h, 37 °C). Following incubation, the supernatants were aspirated, and dimethyl sulfoxide was added (100 μL/well) and incubated at 37 °C for 30 min. The optical density of the formazan product was measured by a microplate reader at 570 nm with a reference wavelength of 690 nm. The IC_50_ values represent the concentration at which 50% cell viability was achieved and offer crucial insights into the cytotoxic effects of the compounds under investigation. The IC_50_ values were determined using GraphPad Prism V5.0 software (GraphPad Software Inc., La Jolla, CA, USA) by fitting the dose–response data to a nonlinear regression model with a variable slope.

##### ATP Quantification

Intracellular ATP levels were assessed using the luminometric Cell Titer-Glo^®^ assay. Cells were dispensed into a white microplate at a concentration of 20,000 cells in 50 μL 0.1 M PBS, and this setup was prepared in triplicate. Subsequently, 20 μL of ATP Cell Titer-Glo^®^ Reagent was added to each well. Following a 30 min incubation at room temperature in darkness, luminescence was measured using a GloMax 96 Microplate Luminometer. The luminescent signal was quantified in relative light units (RLU).

##### Cell Death Biomarkers

Caspase 9, 8, and −3/−7 activities were assessed using the Caspase-Glo^®^ assay. Following the manufacturer’s instructions, the Caspase-Glo^®^ reagent was reconstituted and applied to a 96-well white microplate. Specifically, 20 μL of reagent was added to each well containing 50 μL of a cell suspension (20,000 cells per well), and this setup was replicated in triplicate. Subsequently, the samples were incubated in darkness at room temperature for 30 min. The luminescent signal, indicative of caspase activity, was then quantified using a GloMax 96 Microplate Luminometer.

The LDH cytotoxicity detection kit was utilised to assess cell death or damage. For LDH activity measurement, 100 μL of supernatant was transferred into a 96-well microplate in triplicate. Subsequently, a substrate mixture comprising catalyst (diaphorase/NAD+) and dye solution (INT/sodium lactate) was added to the supernatant, and the reaction was allowed to proceed at room temperature for 25 min. Optical density was then measured at 500 nm using a Multiscan Sky UV/Vis plate reader. The results are presented as mean optical density.

#### 4.3.5. Toxicology Profiling

Toxicological profiles of the compounds were predicted using ProTox 3.0 (AG Preissner; Charité–Universitätsmedizin Berlin), an integrated web-based platform for toxicity prediction. ProTox employs a combination of molecular similarity, pharmacophore modelling, fragment-based propensity analysis, and machine learning algorithms to predict acute toxicity (LD_50_), organ-specific toxicity, toxicity endpoint and tox21 Stress response pathways [27].

#### 4.3.6. Drug-likeness and ADME Properties

The Swiss ADME web tool [28] predicted physicochemical and pharmacokinetic properties relevant to oral bioavailability and drug-likeness. Evaluated parameters included molecular weight, heavy atoms, rotatable bonds, hydrogen bond donors/acceptors, lipophilicity (iLogP), solubility (log S), GI absorption, BBB permeation, P-gp substrate status, and CYP enzyme inhibition. Drug-likeness was assessed via Lipinski’s Rule, bioavailability score, and lead-likeness (Table S2). Canonical SMILES generated by ChemOffice Pro 2015 or PubChem were submitted for ADME predictions.

### 4.4. Statistical Analyses

Biological experiments were conducted as three independent experiments (n = 3) in triplicate. Data was analysed using one-way ANOVA and the Bonferroni test for multiple group comparisons unless otherwise stated—GraphPad Prism v. 5.0 software (GraphPad Software Inc., San Diego, CA, USA). Results were considered statistically significant when *p* < 0.05.

## 5. Conclusions

Evaluating cytotoxicity and antioxidant potential provides valuable insights into the multifaceted nature of phytochemicals from *A. africanum*. The degree of chemical structure similarity between compounds **6**, **7**, and **8** may help explain the observed differences in biological activity. Compounds **6** and **7** differ solely by the unsaturation at the α-β bond, while compound **8** contains two additional methoxy groups at the C-3 and C-3′ positions. The increased lipophilicity of compound **8** compared to **7** may contribute to its higher cytotoxicity. In our study, compound 7 induced canonical apoptosis via the extrinsic pathway, whereas compound **8** led to necrotic cell death. These findings suggest a possible link between certain compounds and the hepatotoxic effects reported in animals consuming *A. africanum*, but this association and the observed cytotoxic activities require cautious interpretation and further validation through in vivo and mechanistic studies. Our results support the need for additional research to better understand the dual bioactivity of these phytochemicals. Future studies should include in vivo efficacy and safety assessments, as well as mechanistic work to clarify potential therapeutic relevance and any toxicological risks. Investigating other properties such as anti-inflammatory or antimicrobial effects may also help to contextualise their broader application potential.

## Figures and Tables

**Table 1:** NMR data of compound **8** in DMSO-*d*_6_.

Position	^δ^C	^δ^H, *multi*, *J*(Hz)
1	136.4	-
2	113.8	7.50 (*br s*)
3	160.1	-
4	117.8	7.00 (*br d*, 8.0)
5	130.4	7.37 (*br t*, 8.0)
6	122.4	7.44 (*br d*, 8.0)
1′	114.0	-
2′	159.0	-
3′	135.2	-
4′	158.4	-
5′	108.7	6.51 (*d*, 8.0)
6′	127.8	8.01(*d*, 8.0)
C=O	192.4	-
α	121.9	7.97 (*d*, 15.6)
β	144.2	7.87 (*d*, 15.6)
3′-OCH_3_	60.2	3.74 (*s*)
3-OCH_3_	55.8	3.83 (*s*)
2′-OH	-	13.55 (*s*)
4′-OH	-	8.02 (*s*)

**Table 2:** Cytotoxicity of compounds **1**–**8** against HepG2 and SH-SY5Y human cancer cell lines (n = 3).

Sample/ Comp.	IC_50_ (µg/mL)	
	HepG2	SH-SY5Y
**1**	NA	71.36
**2**	52.9	265.7
**3**	166.7	172.7
**4**	94.9	228.5
**5**	38.6	115.5
**6**	63.27	99.2
**7**	3.0	47.5
**8**	24.14	19.45
TE	131.3	394.1

**Table 3:** Toxicological profile of the isolated compounds.

Compounds	Predicted LD50 (mg/kg)	Hepatoxicity (Probability)	Neurotoxicity (Probability)	BBB-Toxicity (Probability)	Mitochondrial Membrane Potential (Probability)
**1**	3919	Inactive (0.68)	Inactive (0.86)	Active (0.61)	active (1.0)
**2**	3919	Inactive (0.68)	Inactive (0.86)	Active (0.61)	active (1.0)
**3**	4000	Inactive (0.68)	Inactive (0.86)	Active (0.61)	active (1.0)
**4**	2000	Inactive (0.68)	Inactive (0.86)	Active (0.61)	active (1.0)
**5**	2000	Inactive (0.68)	Inactive (0.86)	Active (0.61)	active (1.0)
**6**	500	Inactive (0.61)	Inactive (0.77)	Active (0.61)	active (0.98)
**7**	3600	Inactive (0.56)	Inactive (0.71)	Active (0.61)	active (0.98)
**8**	2652	Inactive (0.56)	Inactive (0.71)	Active (0.61)	active (0.98)

Physiological, pharmacokinetic, drug-likeness and lead-likeness properties.

## Data Availability

Raw data and data supporting results can be requested from https://www.cput.ac.za/lib, accessed on 27 July 2025.

## Supplementary Materials

The following supporting information can be downloaded at: https://www.mdpi.com/article/10.3390/plants14152389/s1, Table S1: ^1^H- and ^13^C-NMR spectroscopic data (400/100 MHz) for compounds (**1**–**7**); Figure S1: ^1^H NMR of compound **1**. Figure S2: ^1^H NMR of compound **2**. Figure S3: ^1^H -NMR of compound **3**. Figure S4: ^1^H-NMR of compound **4**. Figure S5: ^1^H of compound **5**. Figure S6: ^1^H-NMR of compound **6**. Figure S7: 1H-NMR of compound **7**. Figure S8: 1D and 2D-NMR (400 MHZ, DMSO-*d*_6_) spectra of compound **8**. Figure S9: High-resolution mass spectrometry of compound **8.** Figure S10: IR spectrum of compound **8.** Table S2: Physiological, pharmacokinetic, drug-likeness and lead-likeness properties.
