# Supplementary material for: Bioactive Chalcones from Aizoon africanum: Isolation and Cytotoxicity Against Liver and Neural Cancer Cells
Source: Plants (Basel). 2025 Aug 2;14(15):2389. doi: 10.3390/plants14152389 (PMC12349276; doi:10.3390/plants14152389)
Supplement: Supplementary file 1 [file plants-14-02389-s001.zip › plants-3765646-supplementary.pdf]

Bioactive Chalcones from *Aizoon africanum*: Isolation and Cytotoxicity Against Liver and Neural Cancer Cells

Ali O. E. Eltahir [1,6], Naeem Sheik Abdul[2], Taskeen. F. Docrat[3], Paolo Bristow[2], Elias Chipofya[3], Robert C. Luckay[4], Monde A. Nyila[5], Jeanine L Marnewick[3], Kadidiatou O. Ndjoubi[1] and Ahmed A. Hussein[1]*

Supplementary information

Table S1; ^{1}H- and ^{13}C-NMR spectroscopic data (400/100 MHz) for compounds (**1** – **8**); isolated from *A. africanum*

Figure S1; ^{1}H NMR spectrum (400/100 MHz, Acetone-d_6) of compound **1.**

Figure S2; ^{1}H NMR (400 MHz, DMSO-d_6) spectrum of compound **2.**

Figure S3; ^{1}H-NMR (400 MHz, DMSO-d_6) spectrum of compound **3.**

Figure S4; ^{1}H-NMR (400 MHz, DMSO-d_6) spectrum of compound **4.**

Figure S5; ^{1}H and ^{13}C-NMR (400 MHz, Acetone- d_6 + CD_3OD) spectra of compound **5.**

Figure S6; ^{1}H-NMR (400 MHz, Acetone-d_6) spectrum of compound **6.**

Figure S7; ^{1}H-NMR (400 MHz, DMSO-d_6) spectrum of compound **7.**

Figure S8; 1D and 2D-NMR (400 MHz, DMSO-d_6) spectra of compound **8**.

Figure S9; High-resolution mass spectrometry (HRMS); compound **8**

Figure S10; IR spectrum of compound **8**.

Table S11: Physiological, pharmacokinetic, drug-likeness and lead-likeness properties

Table S1; ^{1}H- and ^{13}C-NMR spectroscopic data (400/100 MHz) for compounds (**1** – **7**); isolated from *A. africanum*

	**1**		**2**	**3**		**4**		**5**	
Position	δ_C	δ_H, *multi, J*	δ_H, *multi, J*	δ_C	δ_H, *multi, J*	δ_C	δ_H, *multi, J*	δ_C	δ_H, *multi, J*
1	-	-	-	-	-	-	-		
2	79.0	5.42 (*dd*, 3.1, 12.7)	5.57 (*dd*, 2.4, 12.5)	78.9	5.54 (*dd*, 2.0, 12.4)	74.3	5.70 (*dd*, 2.8, 13.4)	161.5	
3	42.7	3.01 (*dd* 12.7, 17.0)<br>2.66 (*dd*, 3.1, 17.0)	3.23 (*t*, 12.5, 17.0)<br>2.78 (*dd*, 2.4, 17.0)	42.6	3.24 (*dd* 12.4, 17.2)<br>2.77 (*dd*, 2.0, 17.2)	41.4	3.21 (*dd* 13.4, 17.4)<br>2.71 (*dd*, 2.8, 17.4)	109.6	7.00 (s)
4	195.8	-	-	196.9	-	196.8	-	182.5	-
5	163.5	-	-	158.3	-	163.9	-	162.0	-
6	95.0	5.92 (*d*, 1.5)	-	129.6		96.3	5.89 (*d*, 2.2)	98.6	6.36 (*d*, 2.2)
7	167.4	-	-	160.5	-	167.1	-	164.2	-
8	96.1	5.89 (*d*, 1.5)	5.90 (*s*)	95.6	6.00 (*s*)	95.5	5.91 (*d*, 2.2)	93.7	6.11 (*d*, 2.2)
9	162.2	-	-	155.5	-	163.8	-	188.1	-
10	102.3	-	-	102.2	-	102.1	-	104.3	-
1′	138.7	-	-	139.2	-	125.1	-	117.9	-
2′	126.6	7.52 (*br d*, 7.1)	7.49 (*br d*, 7.1)	127.0	7.50 (*br d*, 7.1)	154.7	-	156.4	-
3′	128.5	7.43 (*t*, 7.1)	7.42 (*t*, 7.1)	129.0	7.43 (*t*, 7.1)	115.9	6.87 (*br d*, 8.0)	116.8	6.93 (*dd*, 1.2, 6.9)
4′	128.5	7.39 (*m*)	7.37 (*m*)	127.0	7.40 (*m*)	129.4	7.21 (*td*, 1.8, 8.0)	132.5	7.27 (*ddd*, 1.2, 6.9, 8.0)
5′	128.5	7.43 (*t*, 7.1)	7.42 (*t*, 7.1)	129.0	7.43 (*t*, 7.1)	119.9	6.86 (*t*, 8)	119.8	6.90 (*ddd*, 1.1, 6.9, 7.1)
6′	126.6	7.43 (*br d*, 7.1)	7.49 (*br d*, 7.1)	127.0	7.50 (*br d*, 7.1)	127.5	7.42 (*br d*, 8.0)	128.6	7.79 (*dd*, 1.2, 7.17))
$-OCH_3$	-	-		64.4	3.68 (*s*)	-		-	
C5-OH		12.02, *s*	12.15		12.17	-	12.02 (*s*)	-	12.78 (*s*)
C7-OH						-	10.07 (*s*)		
C2′-OH							8.70 (*s*)		

Cont. table

Name	6		7	
Position	**δ$_C$**	**δ$_H$, *multi, J,*** Hz	**δ$_C$**	**δ$_H$, *multi, J,*** Hz
1	141.3		135.0	-
2	128.4*	7.12–7.18	129.4*	7.90 (*m*)
3	128.3*		129.3*	
4	125.9	7.04 *m*	131.1	7.46 (*m*)
5	128.3*	7.12–7.18	129.3*	
6	128.4*		129.4*	7.90 (*m*)
1′	112.8		113.4	-
2′	164.9		165.8	-
3′	102.7	6.19 (*d*, 2.5)	103.0	6.29 (*d*, 2.0)
4′	164.9		166.2	-
5′	107.9	6.28 (*dd*, 2.5, 8.7)	108.7	6.41 (*dd*, 8.8, 2.0)
6′	132.7	7.69 (*d*, 8.7)	133.6	8.20 (*d*, 8.7)
(C=O)	203.9		191.9	-
ά	39.0	3.18 (*t*, 7.4)	121.7	7.47 (*d*, 15.5)
β	30.0	2.88 (*t*, 7.4)	144.1	7.98 (*d*, 15.5)
2′-OH	-	12.36	-	13.38 (*s*)

*Exchangeable signals in the same column

Figures S1-S7. ^{1}H-NMR spectra of compounds 1 – 7 isolated from *A. africanum*

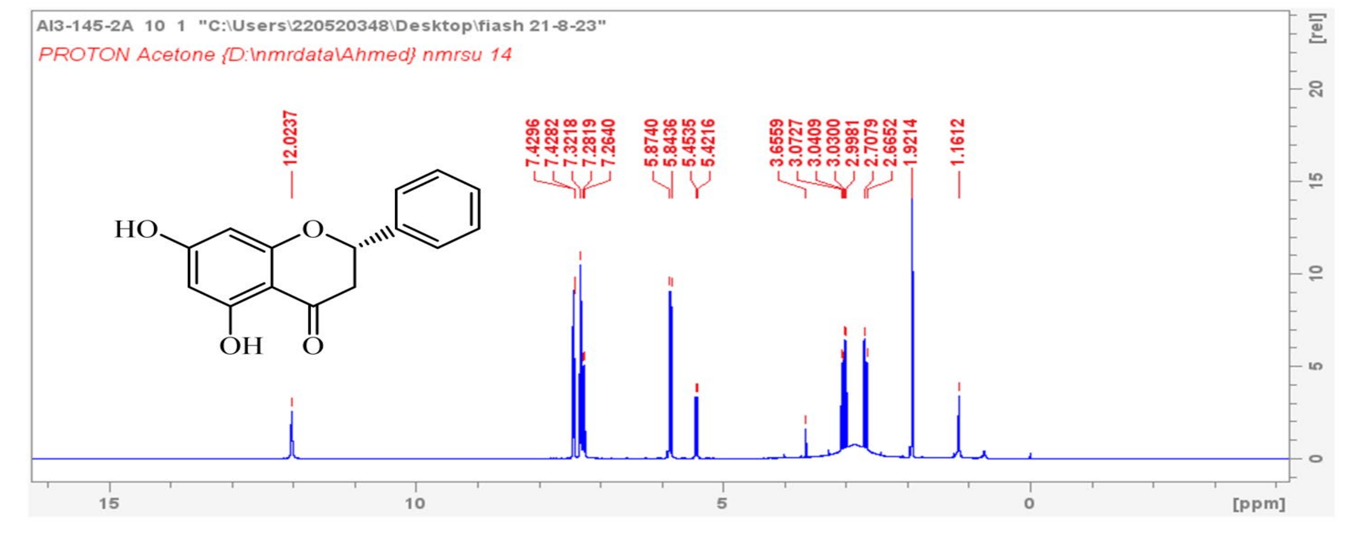

Figure S1; ^{1}H NMR spectrum (400/100 MHz, Acetone-d_6) of compound **1.**

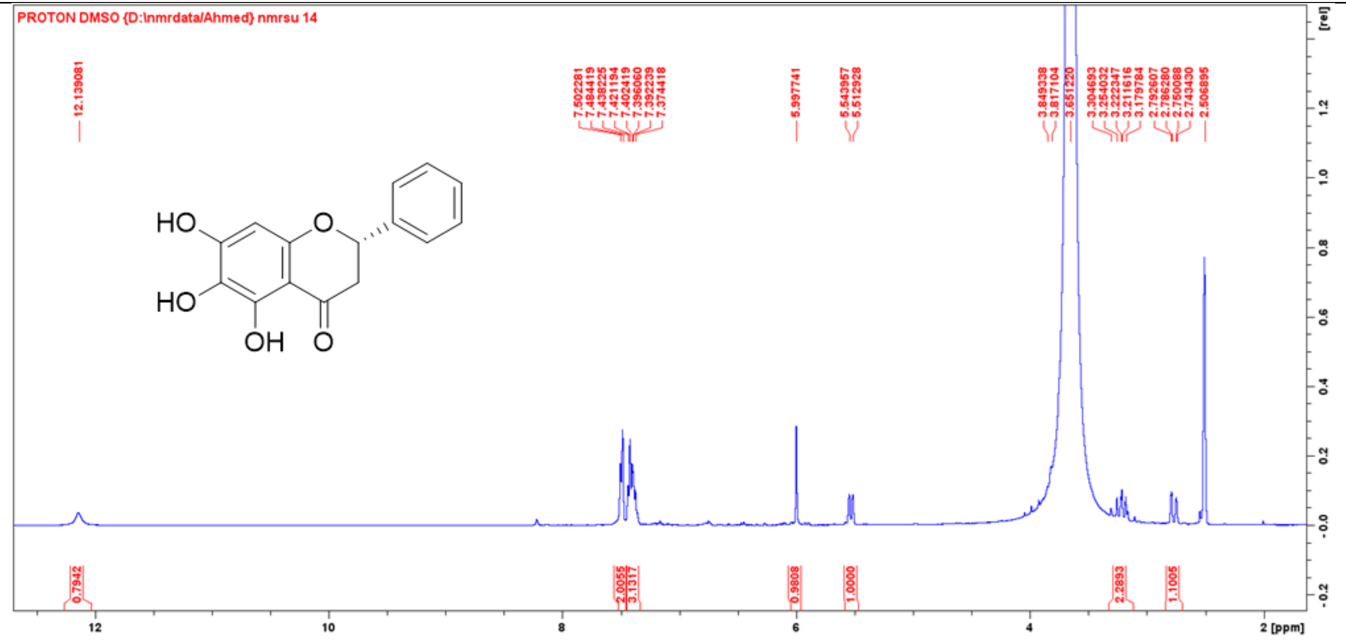

Figure S2; ^{1}H NMR (400 MHz, DMSO-d_6) spectrum of compound **2.**

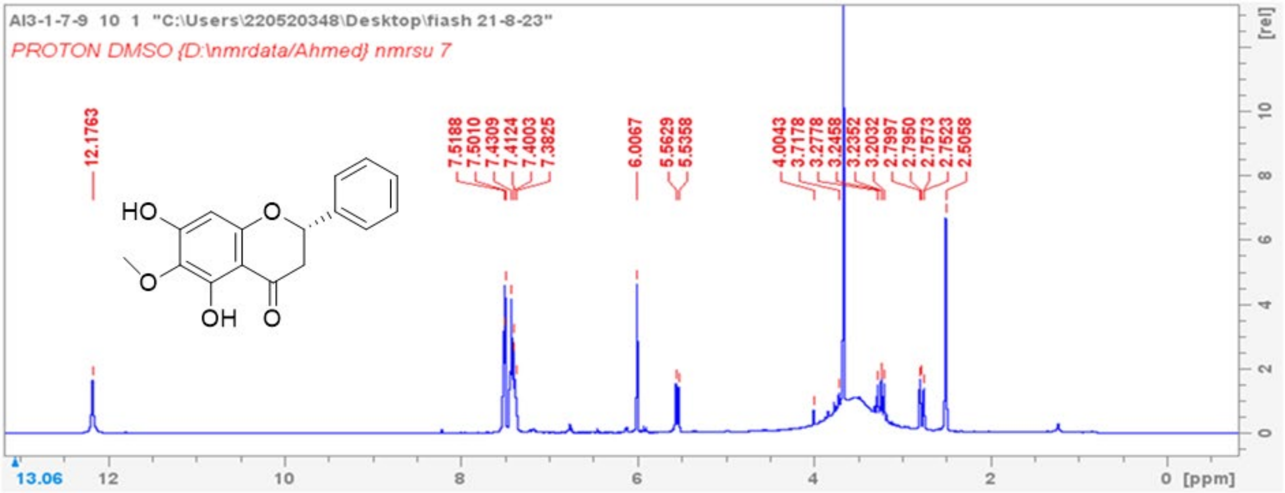

Figure S3; ^{1}H-NMR (400 MHz, DMSO-d_6) spectrum of compound **3.**

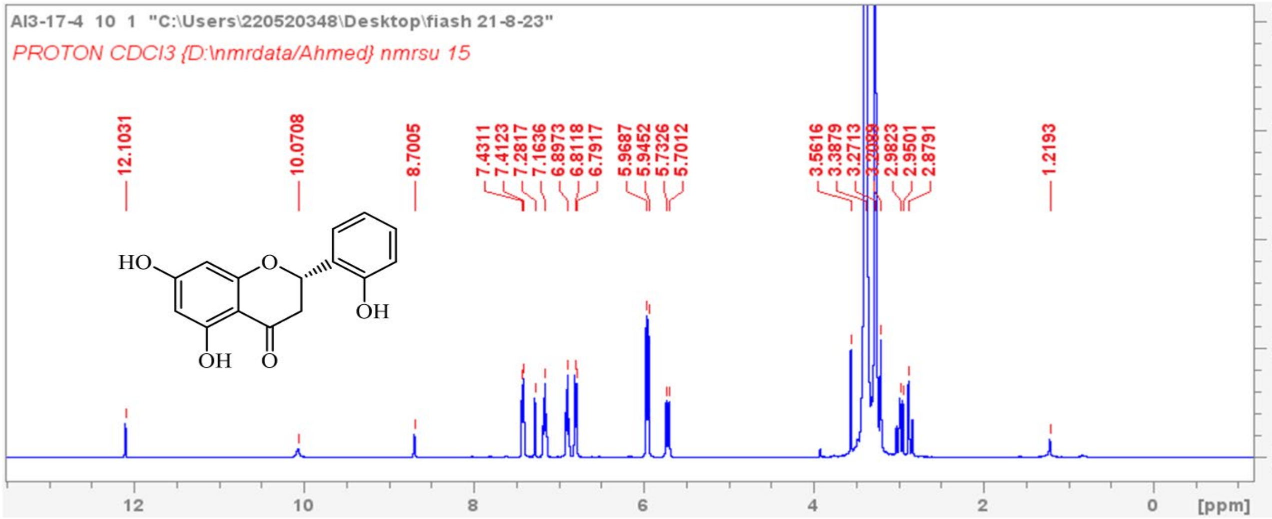

Figure S4; ^{1}H-NMR (400 MHz, DMSO-d_6) spectra of compound **4.**

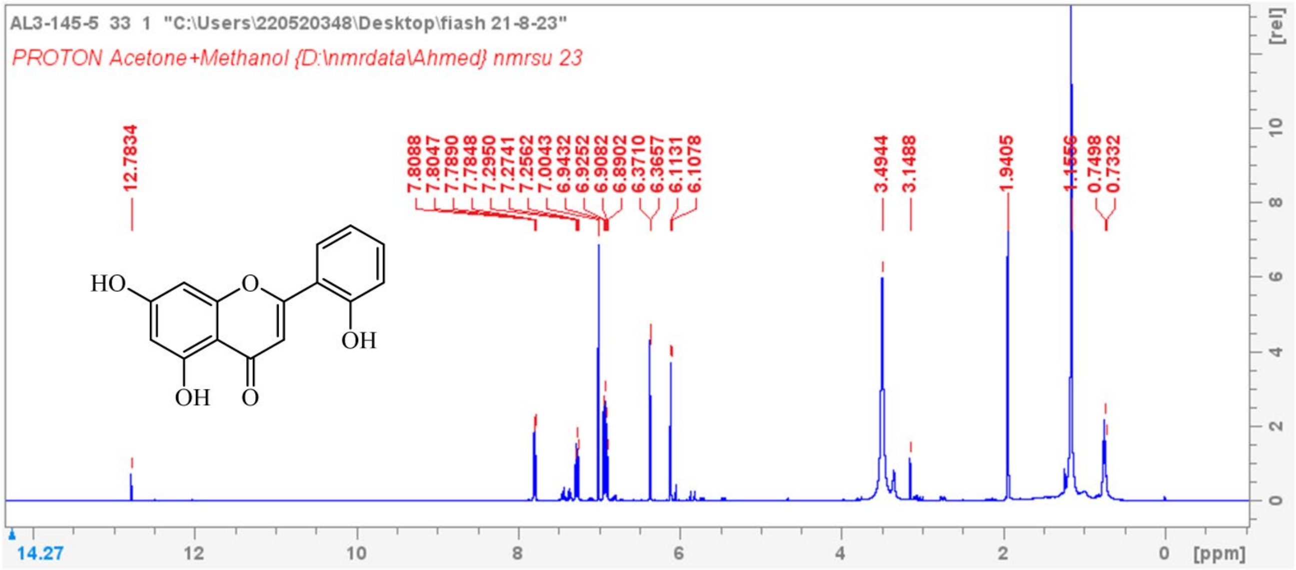

Figure S5; ^{1}H and ^{13}C-NMR (400 MH$_Z$, Acetone- d_6 + CD_3OD) spectrum of compound **5.**

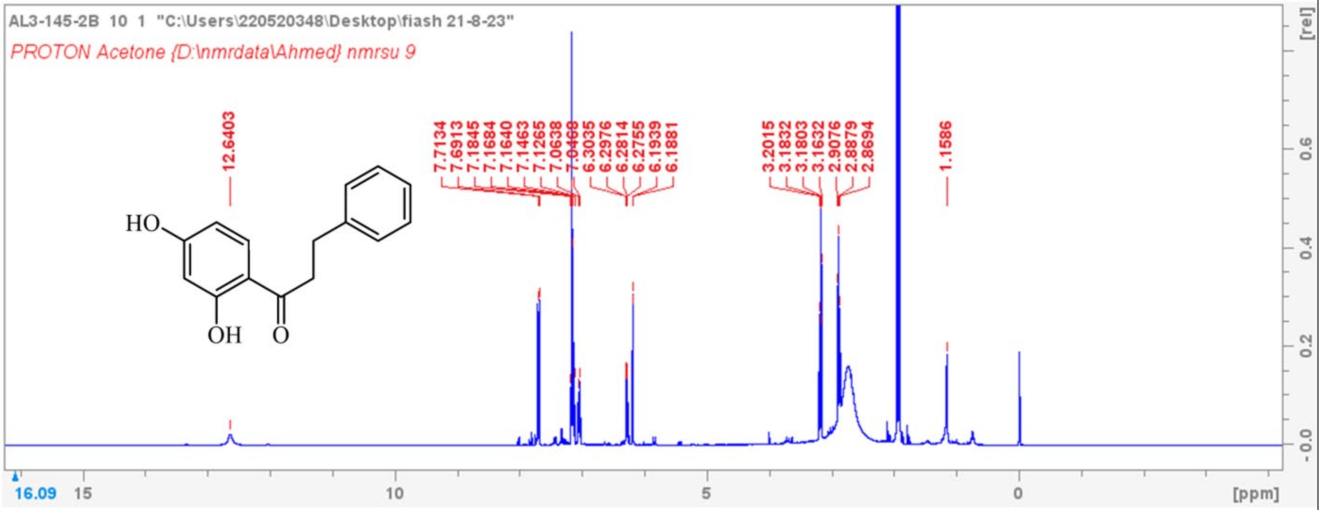

Figure S6; ^{1}H-NMR (400 MH$_Z$, Acetone-d_6) spectrum of compound **6.**

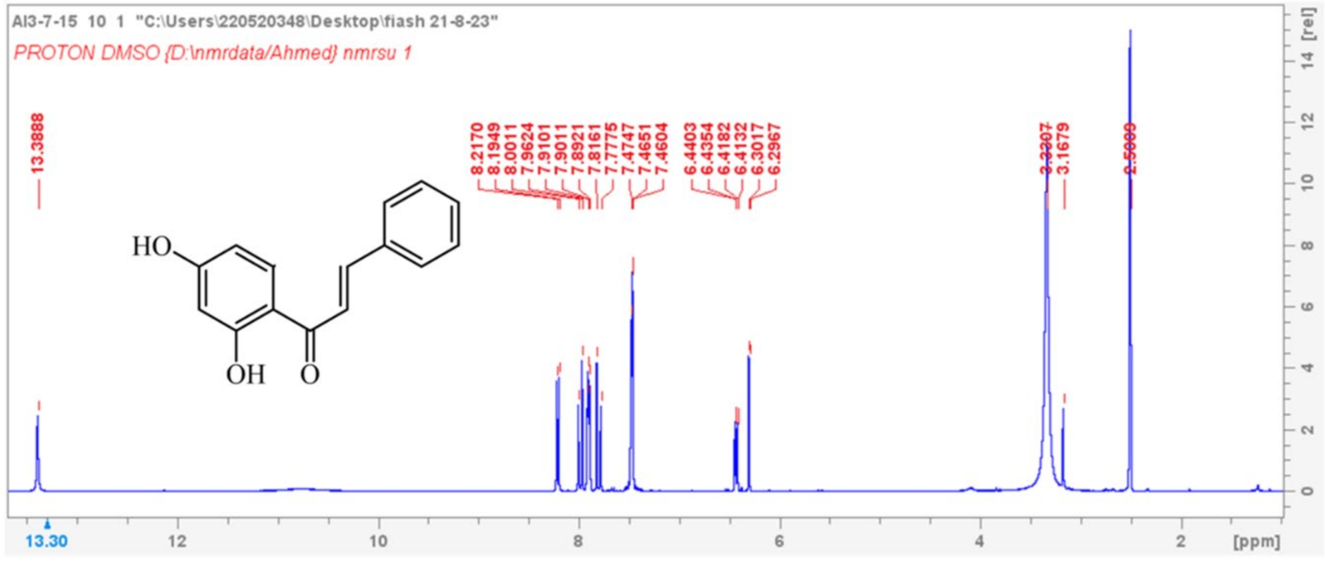

Figure S7; ^{1}H-NMR (400 MH$_Z$, DMSO-d_6) spectrum of compound **7.**

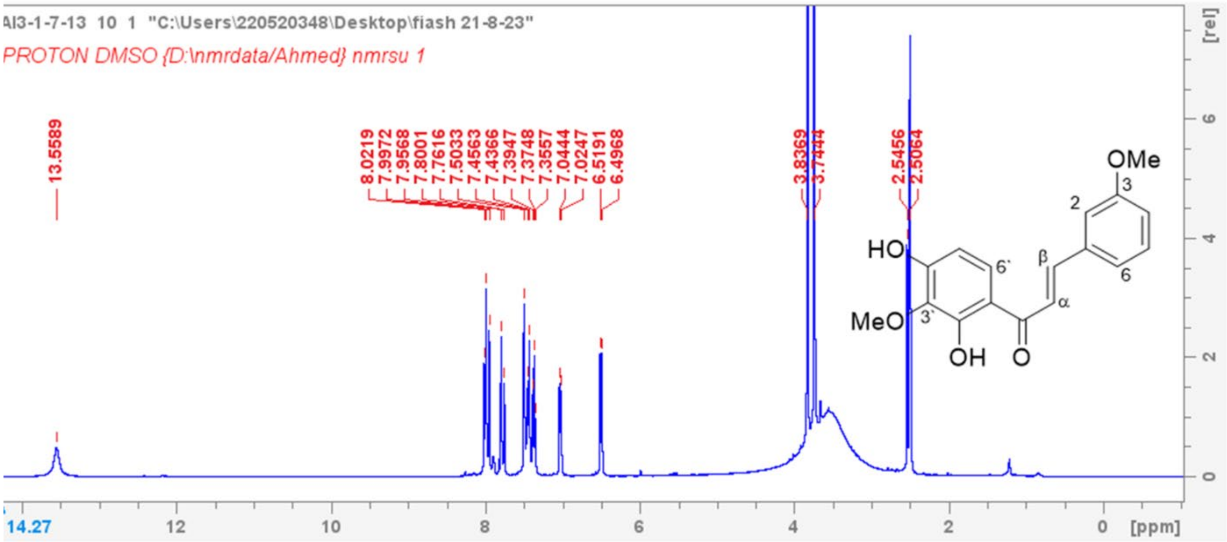

AI3-1-7-13 10 1 "C:\Users\220520348\Desktop\fiash 21-8-23"
PROTON DMSO {D:\nmrdata/Ahmed} nmrsu 1
13.5589
8.0219
7.9972
7.9568
7.8001
7.7616
7.5033
7.4563
7.4366
7.3947
7.3748
7.3557
7.0444
7.0247
6.5191
6.4968
3.8369
3.7444
2.5456
2.5064
OMe
HO
MeO
OH
O
[ppm]
[rel]

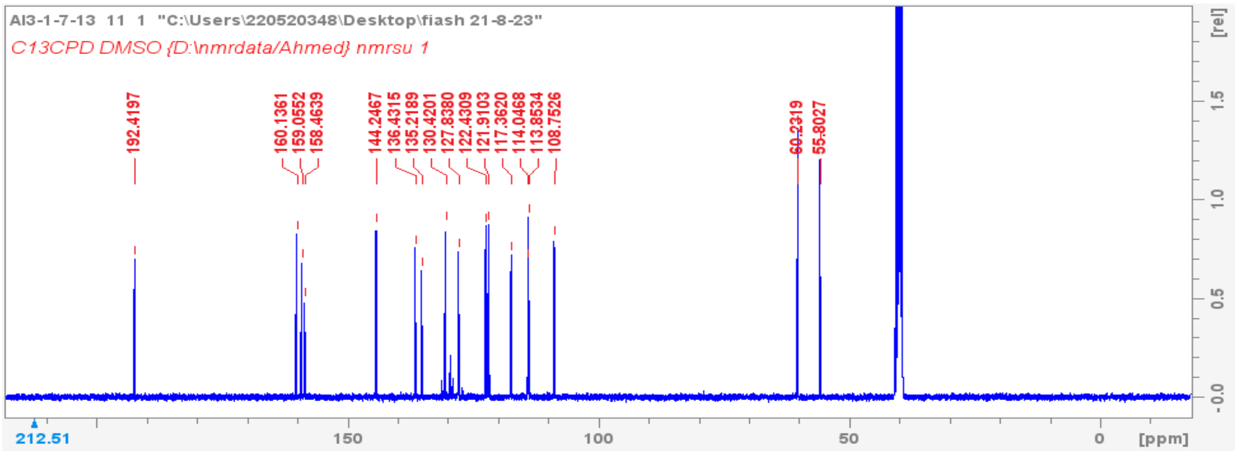

AI3-1-7-13 11 1 "C:\Users\220520348\Desktop\fiash 21-8-23"
C13CPD DMSO {D:\nmrdata/Ahmed} nmrsu 1
192.4197
160.1361
159.0552
158.4639
144.2467
136.4315
135.2189
130.4201
127.8380
122.4309
121.9103
117.3620
114.0468
113.8534
108.7526
60.2319
55.8027
212.51
[ppm]
[rel]

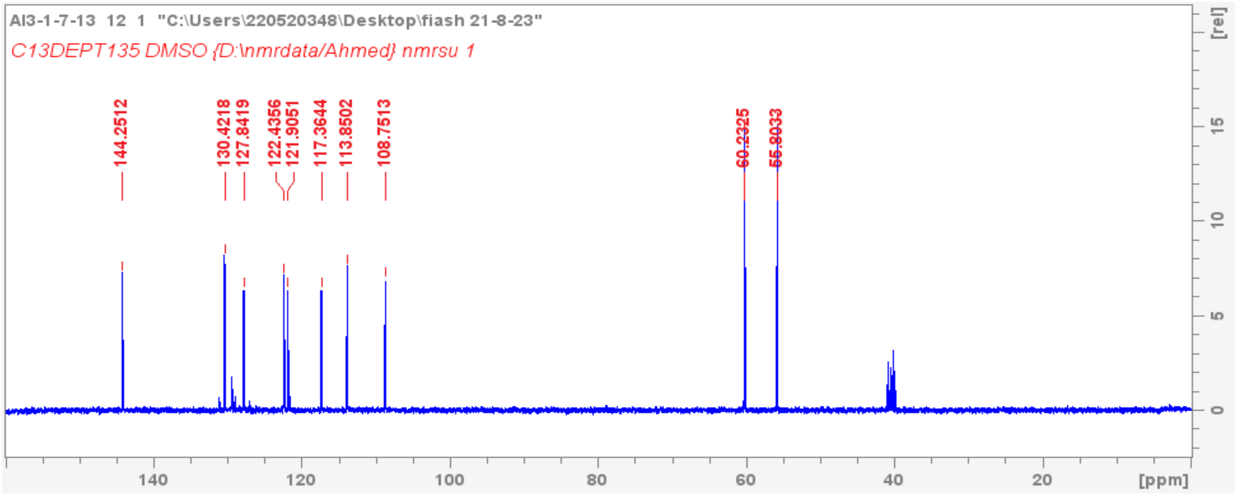

AI3-1-7-13 12 1 "C:\Users\220520348\Desktop\fiash 21-8-23"
C13DEPT135 DMSO {D:\nmrdata/Ahmed} nmrsu 1
144.2512
130.4218
127.8419
122.4356
121.9051
117.3644
113.8502
108.7513
60.2325
55.8033
[ppm]
[rel]

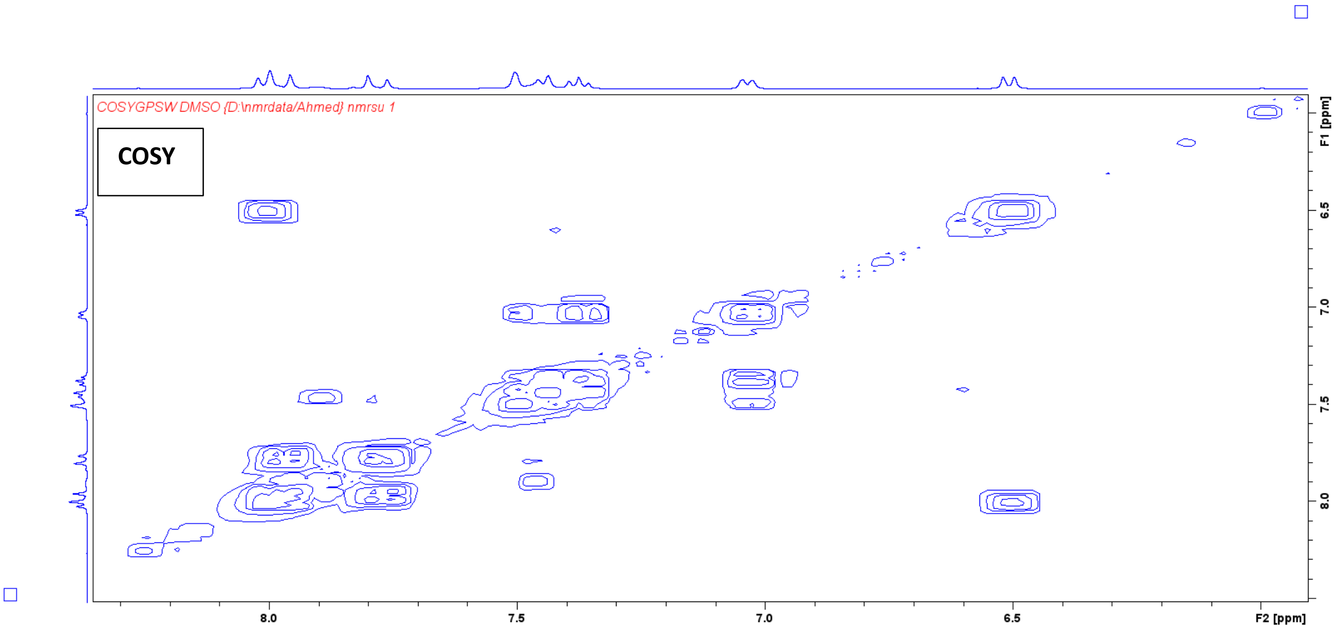
COSYGPSW DMSO {D:\nmrdata/Ahmed} nmrsu 1
COSY
F1 [ppm]
F2 [ppm]

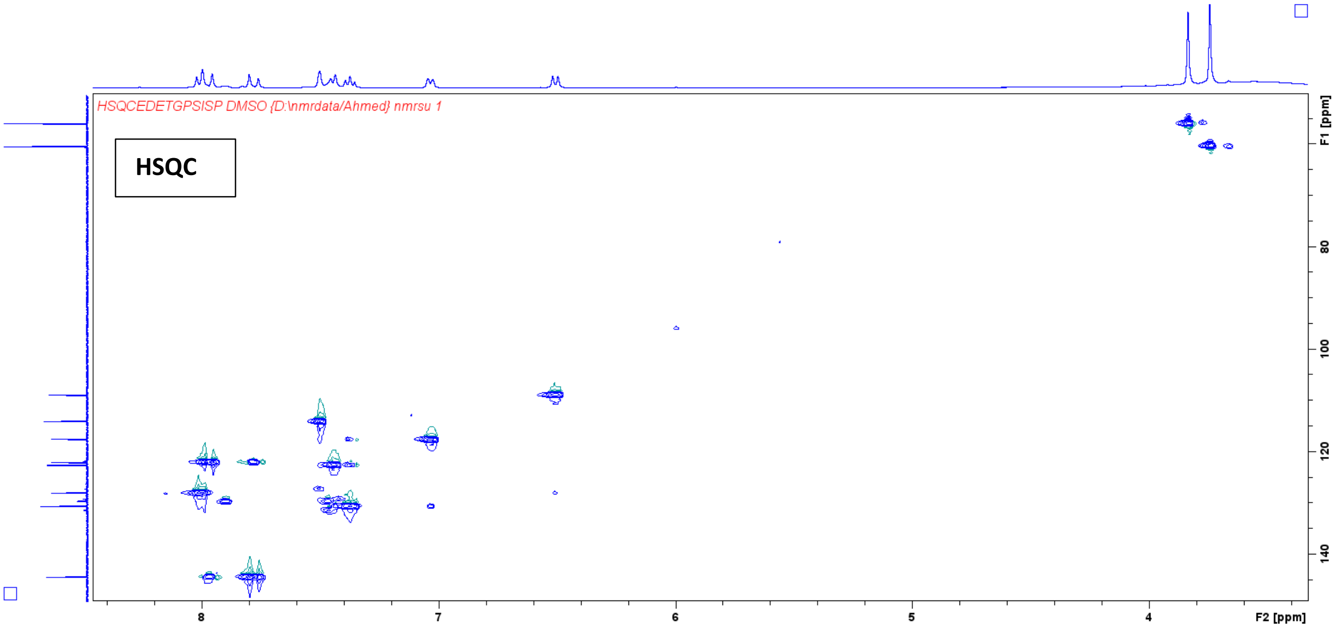
HSQCEDETGPSISP DMSO {D:\nmrdata/Ahmed} nmrsu 1
HSQC
F1 [ppm]
F2 [ppm]

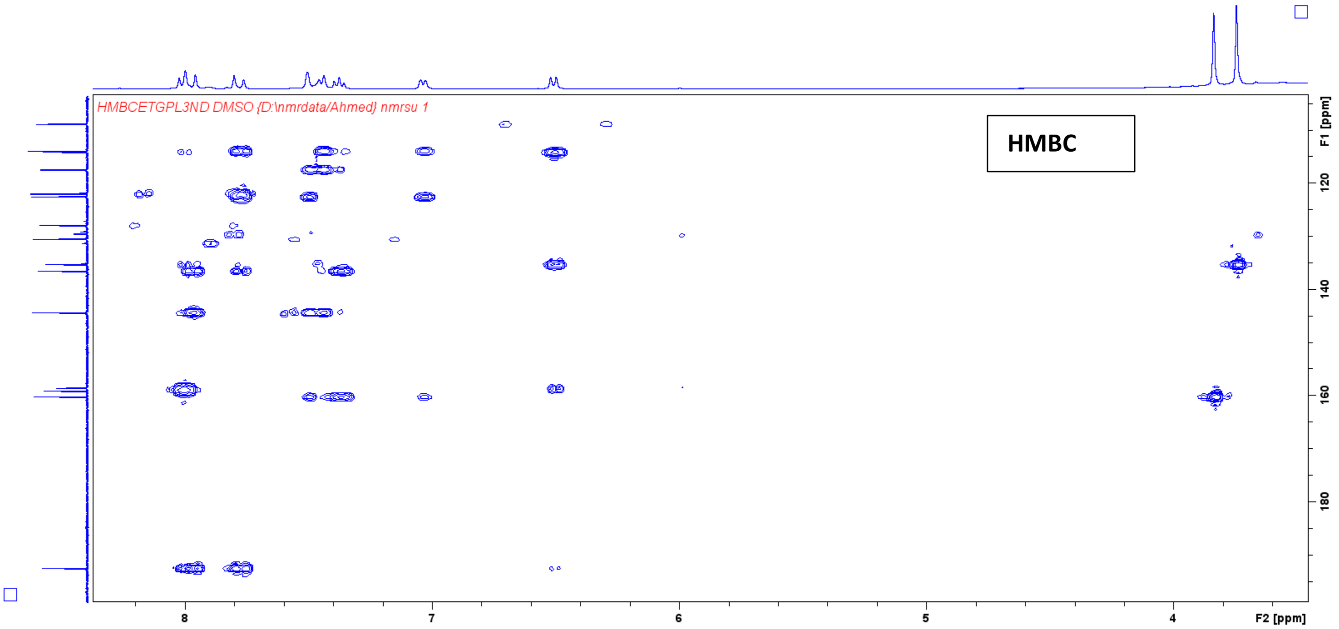

Figure S8; 1D and 2D-NMR (400 MHz, DMSO-d_6) spectra of compound **8**.

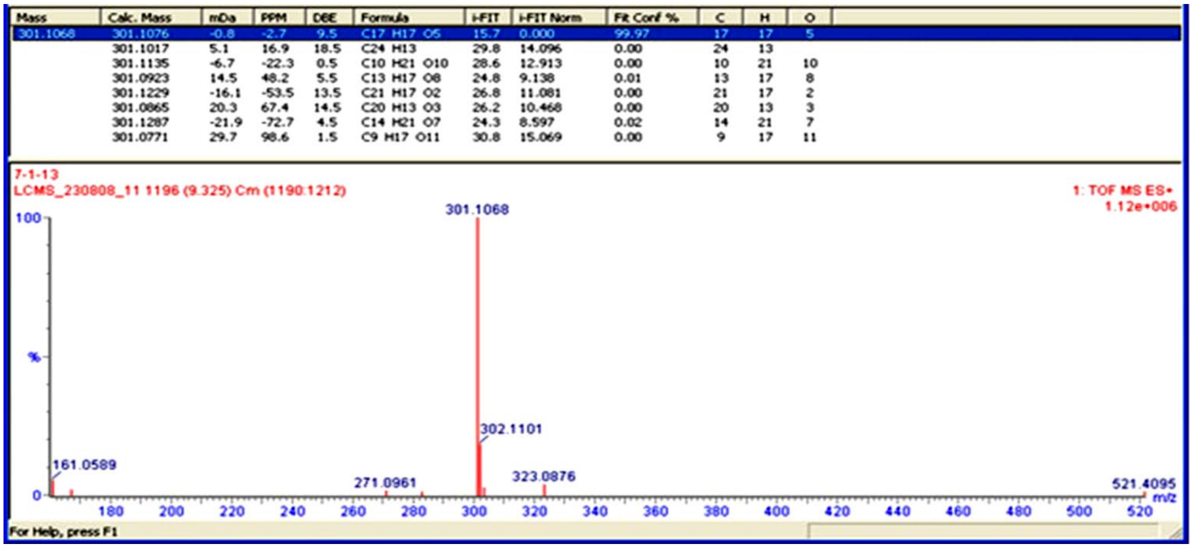

Mass	Calc. Mass	mDa	PPM	DBE	Formula	i-FIT	i-FIT Norm	Fit Conf %	C	H	O
301.1068	301.1076	-0.8	-2.7	9.5	C17 H17 O5	15.7	0.000	99.97	17	17	5
	301.1017	5.1	16.9	18.5	C24 H13	29.8	14.096	0.00	24	13	
	301.1135	-6.7	-22.3	0.5	C10 H21 O10	28.6	12.913	0.00	10	21	10
	301.0923	14.5	48.2	5.5	C13 H17 O8	24.8	9.138	0.01	13	17	8
	301.1229	-16.1	-53.5	13.5	C21 H17 O2	26.8	11.081	0.00	21	17	2
	301.0865	20.3	67.4	14.5	C20 H13 O3	26.2	10.468	0.00	20	13	3
	301.1287	-21.9	-72.7	4.5	C14 H21 O7	24.3	8.597	0.02	14	21	7
	301.0771	29.7	98.6	1.5	C9 H17 O11	30.8	15.069	0.00	9	17	11

Figure S9; High-resolution mass spectrometry (HRMS); compound **8**

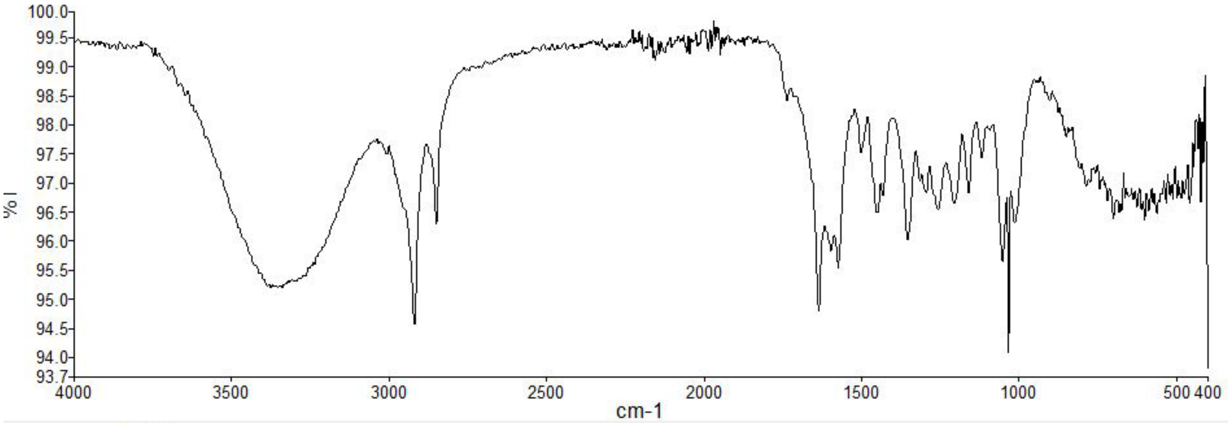

Figure S10; IR spectrum of compound **8**.

Table S11: Physiological, pharmacokinetic, drug-likeness and lead-likeness properties

	**Physiochemical Parameters**							**Lipophilicity**	**Water Solubility**	**Pharmacokinetic Properties**								**Drug Likeliness/ Medicinal Chemistry**		
**Compounds**	**Formula**	**MW (g/mol)**	**HA**	**RB**	**HBA**	**HBD**	**TPSA**	**iLOGP**	**Log S (ESOL)**	**Gi Absorption**	**BBB Permeation**	**P-gp substrate**	**CYP1A2 inhibition**	**CYP2C19 inhibition**	**CYP2C9 inhibition**	**CYP2D6 inhibition**	**CYP3A4 inhibition**	**Lipinski rule**	**Bioavailability score**	**Lead likeness**
**1**	$C_{15}H_{12}O_4$	256.25	19	1	4	2	70.67	2.27	-4.19	High	Yes	No	Yes	No	No	Yes	Yes	Yes; 0 violations	0.55	No ; 1 violation
**2**	$C_{15}H_{12}O_5$	272.25	20	1	5	3	86.99	2.23	-3.49	High	No	Yes	Yes	No	No	No	Yes	Yes; 0 violations	0.55	Yes; 0 violation
**3**	$C_{16}H_{14}O_5$	286.28	21	2	5	2	75.99	2.49	-3.70	High	Yes	No	Yes	No	No	Yes	Yes	Yes; 0 violations	0.55	Yes; 0 violation
**4**	$C_{15}H_{10}O_5$	270.24	16	1	5	3	90.90	1.99	-4.03	High	No	No	Yes	No	No	Yes	Yes	Yes; 0 violations	0.55	Yes; 0 violation
**5**	$C_{15}H_{10}O_5$	270.24	16	1	5	3	90.90	1.99	-4.03	High	No	No	Yes	No	No	Yes	Yes	Yes; 0 violations	0.55	Yes; 0 violation
**6**	$C_{15}H_{14}O_3$	242.27	18	4	3	2	57.53	1.89	-3.68	High	Yes	No	Yes	No	No	No	Yes	Yes; 0 violations	0.55	No; 1 violation
**7**	$C_{15}H_{12}O_3$	240.25	18	3	3	2	57.53	2.23	-3.85	High	Yes	No	Yes	No	Yes	No	Yes	Yes; 0 violations	0.55	No; 2 violations
**8**	$C_{17}H_{16}O_5$	300.31	22	5	5	2	75.99	3.08	-3.85	High	Yes	No	Yes	No	Yes	No	Yes	Yes; 0 violations	0.55	Yes; 0 violation
